# Supplementary material for: Seroprevalence and related risk factors of Brucella spp. in livestock and humans in Garbatula subcounty, Isiolo county, Kenya
Source: PLoS Negl Trop Dis. 2023 Oct 16;17(10):e0011682. doi: 10.1371/journal.pntd.0011682 (PMC10602376; doi:10.1371/journal.pntd.0011682)
Supplement: Attachment — Submitted filename: Reviewed PNTD-D-23-00564.pdf [file pntd.0011682.s003.pdf]

PLOS Neglected Tropical Diseases

Seroprevalence of Brucella spp. in livestock and humans in Isiolo county in Kenya

--Manuscript Draft--

**Manuscript Number:**	PNTD-D-23-00564
**Full Title:**	Seroprevalence of Brucella spp. in livestock and humans in Isiolo county in Kenya
**Short Title:**	Seroprevalence of Brucella spp. in livestock and humans in Isiolo county in Kenya
**Article Type:**	Research Article
**Keywords:**	Brucellosis, human, animals, seroprevalence, risk factors, One Health
**Abstract:**	Background<br>Brucellosis is Brucellosis is a neglected zoonotic disease that affects both animals and humans, causing debilitating illness in humans and socio-economic losses in livestock-keeping households globally. The disease is endemic in many developing countries, including Kenya, but measures to prevent and control the disease are often inadequate among high-risk populations. This study aimed to investigate the human and livestock seroprevalence of brucellosis and associated risk factors of Brucella spp. in a pastoralist region of northern Kenya.<br>Methods<br>A cross-sectional survey was conducted using a two-stage cluster sampling method to select households, livestock, and humans for sampling. Blood samples were collected from 683 humans and 2157 animals, and Brucella immunoglobulin G (IgG) antibodies were detected using enzyme-linked immunosorbent assays. A structured questionnaire was used to collect data on potential risk factors associated with human and animal exposures. Risk factors associated with Brucella spp. exposures in human and livestock were identified using Multivariate logistic regression<br>Results<br>The results indicated a high overall livestock Brucella spp. seroprevalence of 10.4% (95 Confidence Interval (CI): 9.2- 11.7). Camels had the highest exposure rates at 19.6% (95 CI: 12.4-27.3), followed by cattle at 13.2 % (95 CI: 9.3-17.1), goats at 13.1% (95 CI: 11.1-15.3) and sheep 5.4% (95 CI: 4.0-6.9). The herd-level seroprevalence was 51.7% (95 CI: 47.9-55.7). Adult animals (Adjusted Odds Ratio (aOR) =2.3, CI=1.3-4.0), female animals (aOR=1.7, CI: 1.1-2.6), and large herd sizes (aOR =2.3, CI=1.3-4.0) were significantly associated with antibody detection while that sheep had significantly lower odds of Brucella spp. exposure compared to other animal species. Human individual and household seroprevalences were 54.0% (95 CI: 50.2-58.0) and 86.4% (84.0 -89.0), respectively. Significant risk factors associated with human seropositivity included being male (adjusted Odds Ratio(aOR) = 2.1, CI:1.3- 3.2), residing in Sericho ward (aOR= 1.6, CI:1.1-2.5) and having no formal education (aOR=3.0, CI:1.5- 5.9). There was a strong correlation between human seropositivity and herd exposure (aOR=1.6, CI:1.2- 2.3).<br>Conclusions<br>The study provides evidence of high human and livestock exposures to Brucella spp. and identifies important risk factors associated with disease spread. These findings emphasize the need for targeted prevention and control measures to curb the spread of brucellosis and implementation of a One Health surveillance for brucellosis to ensure early detection on the disease in Isiolo County, Northern Kenya.
**Additional Information:**	
**Question**	**Response**
**Government Employee**<br><br>Are you or any of the contributing authors an employee of the United States government?	No - No authors are employees of the U.S. government.

Powered by Editorial Manager® and ProduXion Manager® from Aries Systems Corporation

Manuscripts authored by one or more US Government employees are not copyrighted, but are licensed under a CC0 Public Domain Dedication, which allows unlimited distribution and reuse of the article for any lawful purpose. This is a legal requirement for US Government employees.<br><br>This will be typeset if the manuscript is accepted for publication.	
**Financial Disclosure**<br><br>Enter a financial disclosure statement that describes the sources of funding for the work included in this submission. Review the submission guidelines for detailed requirements. View published research articles from *PLOS Neglected Tropical Diseases* for specific examples.<br><br>This statement is required for submission and **will appear in the published article** if the submission is accepted. Please make sure it is accurate.<br><br>**Funded studies**<br>Enter a statement with the following details:<br>• Initials of the authors who received each award<br>• Grant numbers awarded to each author<br>• The full name of each funder<br>• URL of each funder website<br>• Did the sponsors or funders play any role in the study design, data collection and analysis, decision to publish, or preparation of the manuscript?<br><br>Did you receive funding for this work?	Yes
Please add funding details.<br>as follow-up to "**Financial Disclosure**<br><br>Enter a financial disclosure statement that describes the sources of funding for the work included in this submission. Review the submission guidelines for detailed requirements. View published research	This study was implemented under the project 'Co-infection with Rift Valley fever virus, Brucella spp. and Coxiella burnetii in humans and animals in Kenya: Disease burden and ecological factors' funded by Defense Threat Reduction Agency, Grant No. HDTRA11910031.

articles from *PLOS Neglected Tropical Diseases* for specific examples.<br><br>This statement is required for submission and **will appear in the published article** if the submission is accepted. Please make sure it is accurate.<br><br>**Funded studies**<br>Enter a statement with the following details:<br>• Initials of the authors who received each award<br>• Grant numbers awarded to each author<br>• The full name of each funder<br>• URL of each funder website<br>• Did the sponsors or funders play any role in the study design, data collection and analysis, decision to publish, or preparation of the manuscript?<br><br>Did you receive funding for this work?"	
Please select the country of your main research funder (please select carefully as in some cases this is used in fee calculation).<br>as follow-up to "**Financial Disclosure**<br><br>Enter a financial disclosure statement that describes the sources of funding for the work included in this submission. Review the submission guidelines for detailed requirements. View published research articles from *PLOS Neglected Tropical Diseases* for specific examples.<br><br>This statement is required for submission and **will appear in the published article** if the submission is accepted. Please make sure it is accurate.	KENYA - KE

Powered by Editorial Manager® and ProduXion Manager® from Aries Systems Corporation

**Funded studies**<br>Enter a statement with the following details:<br>• Initials of the authors who received each award<br>• Grant numbers awarded to each author<br>• The full name of each funder<br>• URL of each funder website<br>• Did the sponsors or funders play any role in the study design, data collection and analysis, decision to publish, or preparation of the manuscript?<br><br>Did you receive funding for this work?"	
**Competing Interests**<br><br>On behalf of all authors, disclose any competing interests that could be perceived to bias this work.<br><br>This statement will be typeset if the manuscript is accepted for publication.<br><br>Review the instructions link below and PLOS NTDs' competing interests policy to determine what information must be disclosed at submission.	None
**Data Availability**<br><br>Provide a **Data Availability Statement** in the box below. This statement should detail where the data used in this submission can be accessed. This statement will be typeset if the manuscript is accepted for publication.<br><br>Before publication, authors are required to make all data underlying their findings fully available, without restriction. Review our PLOS Data Policy page for detailed information on this policy. Instructions for writing your Data Availability statement can be accessed via the Instructions link below.	Data is available and accessible

Manuscript

Click here to access/download;Manuscript;Bruc_Seroprev_Draft3_8th May for

Seroprevalence of *Brucella* spp. in livestock and humans in Isiolo county in Kenya

Athman Mwatondo [1,2,3], Mathew Muturi[2,4,5,] James Akoko[2], Richard Nyamota[2], Daniel Nthiwa[6], Josphat Maina[3], Jack Omolo [7], Stephen Gichuhi[8], Marianne W. Mureithi[1], Bernard Bett [2]

1. Department of Medical Microbiology and Immunology, University of Nairobi, Nairobi, Kenya

2. International Livestock Research Institute, Nairobi, Kenya

3. Zoonotic Disease Unit, Ministry of Health, Nairobi, Kenya

4. Dahlem Research School of Biomedical Sciences, Department of Veterinary Medicine, Freie Universität Berlin, Berlin, Germany

5. Zoonotic Disease Unit, Ministry of Agriculture, Livestock and Fisheries, Nairobi, Kenya

6 Department of Biological Sciences, University of Embu, Embu, Kenya

7. County Government of Kilifi, Department of Agriculture, Livestock Development and Fisheries

8. Department of Ophthalmology, University of Nairobi, Nairobi, Kenya

*Corresponding author: Athman Mwatondo

Abstract

Background

Brucellosis is Brucellosis is a neglected zoonotic disease that affects both animals and humans, causing debilitating illness in humans and socio-economic losses in livestock-keeping households globally. The disease is endemic in many developing countries, including Kenya, but measures to prevent and control the disease are often inadequate among high-risk populations. This study aimed to investigate the human and livestock seroprevalence of brucellosis and associated risk factors of *Brucella* spp. in a pastoralist region of northern Kenya.

Methods

A cross-sectional survey was conducted using a two-stage cluster sampling method to select households, livestock, and humans for sampling. Blood samples were collected from 683 humans and 2157 animals, and *Brucella* immunoglobulin G (IgG) antibodies were detected using enzyme-linked immunosorbent assays. A structured questionnaire was used to collect data on potential risk factors associated with human and animal exposures. Risk factors associated with *Brucella* spp. exposures in humans and livestock were identified using Multivariate logistic regression

Results

The results indicated a high overall livestock *Brucella* spp. seroprevalence of 10.4% (95 Confidence Interval (CI): 9.2- 11.7). Camels had the highest exposure rates at 19.6% (95 CI: 12.4-27.3), followed by cattle at 13.2 % (95 CI: 9.3-17.1), goats at 13.1% (95 CI: 11.1-15.3) and sheep 5.4% (95 CI: 4.0-6.9). The herd-level seroprevalence was 51.7% (95 CI: 47.9-55.7). Adult animals (Adjusted Odds Ratio (aOR) =2.3, CI=1.3-4.0), female animals (aOR=1.7, CI: 1.1-2.6), and large herd sizes (aOR =2.3, CI=1.3-4.0) were significantly associated with antibody detection while that sheep had significantly lower odds of *Brucella* spp. exposure compared to other animal species. Human individual and household seroprevalences were 54.0% (95 CI: 50.2-58.0) and 86.4% (84.0 -89.0), respectively. Significant risk factors associated with human seropositivity included being male (adjusted Odds Ratio(aOR) = 2.1, CI:1.3- 3.2), residing in Sericho ward (aOR= 1.6, CI:1.1-2.5) and having no formal education (aOR=3.0, CI:1.5- 5.9). There was a strong correlation between human seropositivity and herd exposure (aOR=1.6, CI:1.2- 2.3).

Conclusions

The study provides evidence of high human and livestock exposures to *Brucella spp.* and identifies important risk factors associated with disease spread. These findings emphasize the need for targeted prevention and control measures to curb the spread of brucellosis and implement a One Health surveillance to ensure early detection of the disease in Isiolo County, Northern Kenya.

Author Summary

Brucellosis is a priority zoonotic disease in Kenya that causes human illness and socio-economic losses in livestock-keeping households. We conducted a linked human-livestock study to investigate the seroprevalence of Brucella spp. in humans and livestock. We also identified risk factors associated with brucellosis seropositivity. The study found a high seroprevalence in humans and animals and identified significant risk factors associated with livestock exposure, which included adult animals, female animals, and large herd sizes. In humans, significant risk factors included being male, residing in a specific ward, and having no formal education. We also found a strong correlation between human seropositivity and herd exposure. Implementing interventions such as promoting public awareness about the disease, safe handling of livestock and livestock products, and livestock vaccinations can potentially reduce the disease transmission in livestock and spill over to humans. Future studies should examine the efficacy of measures that reduce these risk factors to mitigate the spread of brucellosis.

Introduction

Brucellosis is a neglected zoonotic disease that causes debilitating illnesses in humans and extensive socio-economic losses in livestock-keeping households globally(1,2). Six classical *Brucella* species infect animals, but only four have zoonotic potential; *B. abortus,* whose primary host is cattle; *B. melitensis,* which infects goats and sheep and *B. suis* and *B. canis,* which infects pigs and dogs respectively (2–4). Domesticated animals, such as cattle, sheep, goats, camels, and pigs, are the primary sources of human infections, while wild animals may act as reservoirs in areas with livestock-wildlife interactions (5). Human infections are primarily attributed to *B.*

melitensis, the most virulent species(6). Human exposure is principally through consuming unpasteurized milk and dairy products, undercooked meat and meat products and direct contact with an infected aborted fetus, placenta, fetal fluids, and vaginal discharges (7). High-risk groups include livestock keepers, field animal health workers, abattoir workers, dairy industry workers, and laboratory professionals (8,9). Human-to-human transmission of brucellosis is rare, but cases have been reported through transplacental infection, breastfeeding, and organ transplantation (10).

Brucellosis is a systemic disease in humans, mainly presenting as a febrile illness with acute, subacute, or chronic forms depending on the stage of the disease (11,12). The disease is characterized by recurrent, undulant fevers, arthralgia, myalgia, malaise, sweating, lower back pain and headache (8,13). The symptoms are often accompanied by localized single or multiple-organ involvement (12). Due to the unspecific nature of the signs and symptoms of brucellosis, diagnosis requires assessment of a combination of clinical, epidemiological and laboratory criteria. Treating brucellosis is costly, as it often requires a prolonged course of antibiotic therapy. Furthermore, treatment may be complicated by factors such as treatment failure, relapses, and severe side effects from the drug regimen (12). The disease is rarely fatal, with a less than two percent fatality rate in untreated cases. However, if left untreated or inadequately treated, it can lead to long-term consequences such as chronic infections, infertility, arthritis, endocarditis, and neurological complications(14).

Transmission in livestock is mainly through ingestion of pastures or water-contaminated by-products of abortion or parturition like placental membranes and vaginal fluids (15,16). Infection in most livestock species presents as a reproductive disorder characterized by infertility, abortion, reduced newborn survival rates, and decreased milk production. As such, the disease is associated with significant direct economic losses to farmers and indirect losses in endemic countries due to restricted trade in livestock and livestock products (17–19).

Brucellosis has been controlled in most developed countries but is still prevalent in Asia, Africa, Central and South America, and the Middle East. (20), with approximately 500 million human infections reported annually. The incidence is much higher, with estimates ranging from 10 to 25 times the reported numbers(20). In Kenya, brucellosis is endemic and ranked the fourth most important zoonotic disease (21). However, its actual burden on livestock and human populations remains inadequately described due to weak surveillance, low utilization of health care services, and the widespread use of non-prescribed antibiotics among affected communities (23–25). Studies have demonstrated that the disease has been prevalent in pastoralist communities in the last ten years, with seroprevalence estimates ranging from 10 to 47 per cent in apparently healthy humans and 3.4 to 22.3 per cent in livestock(22–26).

The prevention and control of brucellosis in endemic low- and middle-income countries (LMICs) require integrated one-health approaches, includingntegrated research activities, surveillance, and response (27–29). This study was conducted at the interface between humans and livestock in Isiolo County as part of a larger investigation into co-exposures of Rift Valley fever virus, *Brucella* spp, and *Coxiella burnetii* in both humans and animals in northern Kenya. Our objectives were two-fold; to estimate the seroprevalence of *Brucella* spp. and to determine the risk factors

associated with *Brucella* spp. seropositivity. The results of this study will provide valuable evidence and baseline data for establishing an integrated livestock-human surveillance system in the study area.

Materials and methods

Ethics Statement

We sought and obtained ethical approval for human and livestock sampling from the Institutional Research Ethics Committee at the International Livestock Research Institute (reference number: ILRI-IREC2020-07). The household heads provided written consent for livestock sampling, and written consent was obtained from all selected humans for sampling. The study clinicians informed all study participants about the study that their participation was voluntary, and they could decline without any consequences. For adults (aged >18 years), written consents were provided; for children aged between 13–17 years, written assent forms and parental/guardian permissions were obtained. For children under 13 years, only written parental/guardian permissions were obtained, but the children were informed about the study procedures.

Study area

The study was conducted in Garbatula subcounty in Isiolo County. Isiolo County is in the northern region of Kenya (Figure 1). The subcounty is subdivided into three administrative wards: Garbatulla, Kinna and Sericho. The study area is in a relatively low-lying region, with Kinna ward having the highest altitude of 589 meters (m) and the lowest area being Sericho at 459m. The area receives an average of 580 millimetres of rainfall per annum, with November and April being the wettest months. The area is characterized by hot and dry climate for most of the year, with a mean annual temperature of 29^{0}C, with variations due to differences in altitude (30).

Garbatula subcounty is sparsely populated, with an average population density of nine people per square kilometre(31). The Borana tribe is the most predominant, and livestock is a source of livelihood for over 80% of the inhabitants(32). Cattle, sheep, goats, and camels are the main livestock species raised in the area, although goats and sheep have the highest population. Garbatula subcounty was purposefully selected for the study because of good accessibility, better security, and its rich wildlife diversity; the subcounty shares a border with Meru National Park and the Bisanadi Game Reserve, two unconfined sanctuaries for wild animals. As a result, there are frequent interactions between domestic livestock and wild animals in certain regions of the subcounty.

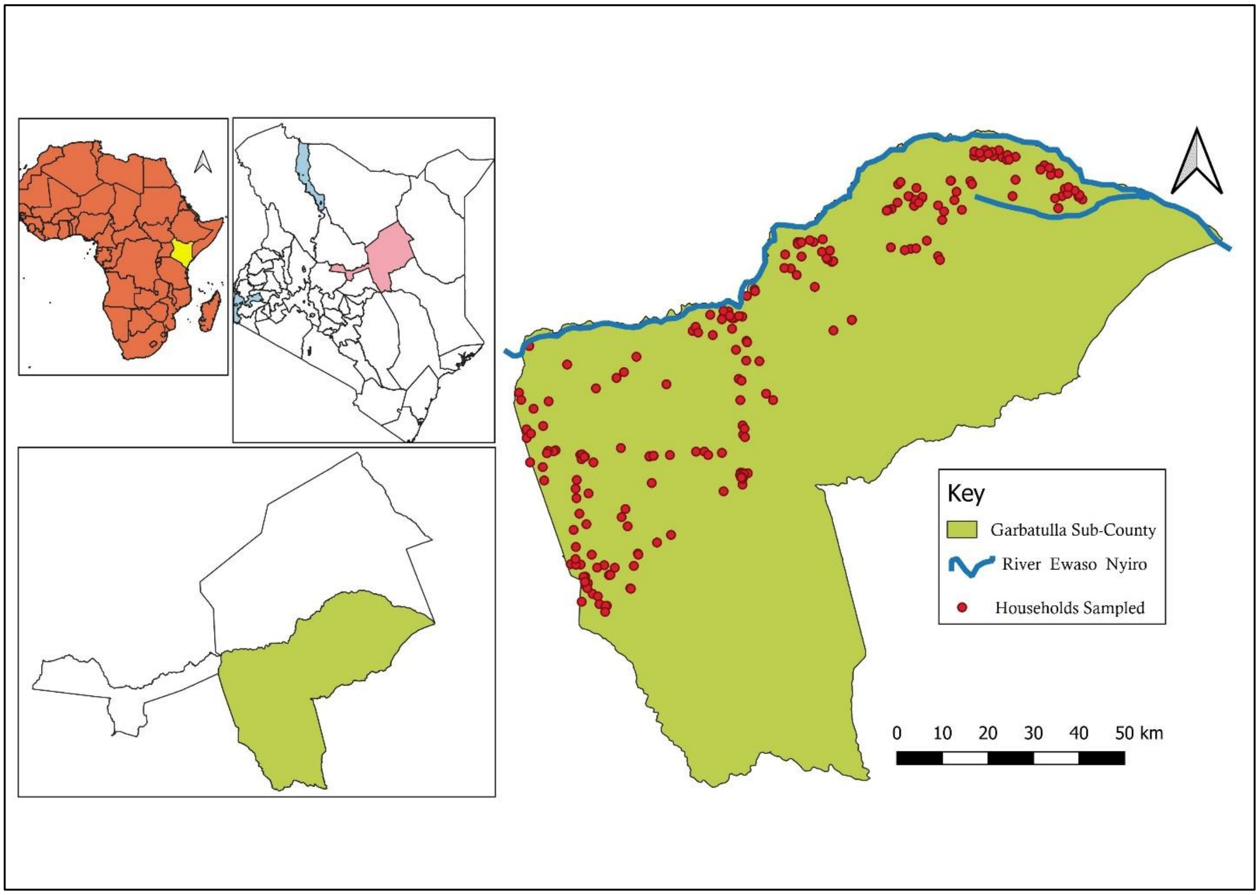

Figure 1: Map of Africa and Kenya showing sampling sites within Garbatula subcounty (*The underlying map layer used to generate this figure was obtained from https://www.diva-gis.org/gdata.*)

Sample size determination

The sample size(n) for humans and animals (camel, cattle, sheep, and goats combined) was estimated using the formula; $n = \frac{1.96^2 * p(1-p)}{d^2}$ (33). The expected (*a priori*) seroprevalence estimate(p) of *Brucella* species was assumed to be 50% in both humans and animals since no reliable references on *Brucella* burden were available from the area. It was further assumed that the study would estimate this outcome with an error margin (d) of 0.05.

Previous studies have shown that *Brucella* spp. seroprevalence clusters in humans and animals at the household and herd levels. A design effect (Deff) was therefore determined for adjusting the naïve sample sizes by assuming that an average of twenty animals per herd and three humans per household would be sampled and that *Brucella* spp. seroprevalences have intracluster correlation coefficients (ICC) of 0.2 in animals and 0.21 in humans, respectively (23). DEFF estimates of 4.8 and 1.42 were derived for animals and humans, respectively. Minimum sample

sizes of 546 humans and 1844 animals were therefore estimated based on multiplying the DEFF estimates with the naïve sample sizes.

Sampling design for livestock and humans

Livestock and human data were collected in July-August 2021 in a cross-sectional, two-stage random sampling design. The primary sampling units were individual livestock and humans within randomly selected households. Households were eligible for selection if they kept or owned any of the four livestock species and had at least one consenting adult human. To identify study households, 250 random geographical coordinates (RGC) were generated using Quantum Global Information System (QGIS) version 3.4.4 software (https://qgis.org), for Garbatula Subcounty and households closest to an RGC were selected for sampling. For the coordinates that fell near a cultural homestead (Manyatta) where livestock from different households are kept in one enclosure, one household was randomly selected, and only humans and animals owned by the household were eligible for sampling.

A household was defined as a group using a common cooking area. A total of five animals were selected per species (camel, sheep, cattle, and goats) in each herd using systematic random sampling. Animals under one month and very sick were excluded from the study. All household members were eligible for enrolment, excluding children under two years since our ethical approval did not cover these.

Sample collection in livestock and humans

Qualified veterinarians collected livestock samples. During sampling, animals were restrained by trained animal handlers. Using aseptic techniques, blood was collected from the jugular vein of each animal into 10 ml plain vacutainers with unique barcode labels. The vacutainers were stored in cool boxes with icepacks to maintain temperatures of 4-8°C in the field before transfer to the local field veterinary laboratory for serum extraction and temporary storage at -20 ^{0}C. The samples were centrifuged at 2500 gravitational force for 15 minutes, and the serum was stored in 2 ml barcoded cryovials. Extracted serum samples were transported at -20c using motorized freezers to the International Livestock Research Institute (ILRI) Laboratories in Kenya every five days, where they were first stored at -20 ^{0}C before testing for antibodies to *Brucella* species and then transferred to -80°C for long-term storage. Qualified clinicians conducted the sampling in humans. Each participant had 5 mL of blood drawn from their median cubital vein on the left hand using plain vacutainers with unique barcode labels. All blood samples were immediately placed in a cool box and maintained at 4-8°C in the field before being transferred to nearby health centres, within four hours. The procedures for extracting serum, storing and transporting samples were identical to those used for animal samples.

Data collection

Data was collected by the trained study staff using standardized questionnaires installed in Open Data Kit (ODK) electronic data collection tool. The questionnaires were uploaded to smartphones

and were pretested in the field before deployment. Clinicians administered household and individual human questionnaires, while veterinarians administered the herd-level and individual animal questionnaire. The first questionnaire was administered to household heads to collect herd-level animal husbandry practices linked with the transmission of *Brucella* spp. such as herd size, breeding between herds, sharing of grazing areas and sale or introduction of new animals into the herd. Individual-level animal data of the recruited animals were then collected, including age, sex, species (cattle, camel, goat, or sheep) and history of reproductive syndromes (abortion, weak birth, stillbirth, infertility, and swollen testis).

Livestock species were categorized into different age groups based on their age range. The first category included suckling animals such as sheep and goats 0-3 months old and cattle and camels 0-6 months old, and these animals usually stay close to households. The second category included weaners or young adults no longer dependent on milk and often separated from their mothers. This category had goats and sheep that were 7-12 months old and cattle and camels that were seven months to two years old (for cattle) and up to three years old (for camels). Animals that were above these age ranges were categorized as adult animals that are of reproductive age.

Lastly, human subject-level data such as demographic information, history of febrile illness, consumption of raw milk and milk products, handling of animals and animal products and occupational practices (herding, milking, slaughtering, and assisting in animal births) were collected.

Laboratory testing

Livestock serum samples were tested using the ID Screen Brucellosis Serum Indirect Multi-species kit (Grabels, France) for the detection of anti-*Brucella* spp. IgG antibodies against in bovine, ovine, caprine, and porcine samples. Briefly, the 10 µl of serum samples were diluted using 190 µl of dilution buffer two and loaded onto the pre-coated ELISA reaction plate. The test plates were incubated at room temperature for 45 minutes, after which they were washed three times. This was followed by the addition of 100 µl of the conjugate solution before the plate was incubated at room temperature for 30 minutes. After three washes, 100 µl of the substrate solution was added, and the plate was incubated in darkness for 15 minutes. The reaction was stopped using 100 µl of the stop solution, and immediately, the optical densities (ODs) of each well were determined at 450 nm wavelength. All the samples were run in duplicates with each plate comprising positive and negative control. The results for each plate were considered valid if the mean OD for the positive control was greater than 0.35 and the ratio of the OD values between the positive and negative controls was greater than 3 . The percentage S/P was calculated for each sample as described by the manufacturer. Following the manufacturer’s recommended cut-off values, all samples whose percentage S/P was greater than or equal to 120% were considered positive, negative if less than or equal to 110% and doubtful if greater than 110% but less than 120%.

All human serum samples were tested using IBL-America IgG enzyme-linked immunosorbent assay (ELISA) kits (IBL-America, Minneapolis, USA) according to the manufacturer's recommendation. Briefly, the sera were diluted at 1:100 using sample diluent and added onto antigen pre-coated microtitre plates followed by incubation for 1 hour at 37°. Thereafter, 100 µl of ready to use conjugate was added and incubated for 30 minutes at 37°C before adding para-Nitrophenyl Phosphate (pNPP)substrate and further incubated at 37°C for 30. The conjugate–substrate reaction was done by adding 100 µl of ready-to-use stop solution included in the kit. The test sera ODs were read at 405 nm against a substrate blank and 630 nm was used as a reference wavelength to correct for the background fluorescence. Wash steps preceded both conjugate and substrate addition steps, while all sera and controls were run in duplicates. The results for each plate were valid when the substrate blank OD was less than 0.25 and the negative control gave negative results as per the interpretations. We first calculated the upper and lower cut-off limits to interpret our results. The upper cut-off limit was calculated as a product of the serum OD, the kit-specific correction factor (0.666) and the kit-specific grey zone given as 14% of the mean standard OD divided by Positive control OD). To calculate the lower cut-off limit, the kit specific grey zone was subtracted from the product of sample OD and the kit specific correction factor. Samples were interpreted as positive if their mean OD fell above the upper cut-off limit, doubtful if between the upper and lower cut-off limits, and negative if below the lower cut-off limit. Doubtful samples were considered as negative in subsequent analyses.

Data management and statistical analyses

The serological and questionnaire data were first combined into one comma-delimited value (.CSV) file and cleaned prior to analysis. We performed all analyses using the R statistical environment, version 3.6.0 (34), with descriptive statistics being analyzed using the *gmodels* (35) and *epiR* (36) packages. The first analysis was the calculation of animal/subject and household/herd levels overall seroprevalence estimates of *Brucella* spp., with a 95% confidence interval (CI) in humans and livestock. Seroprevalence was assessed at the individual, household, and herd levels. A household or herd was considered seropositive if at least one human or animal, respectively, tested positive. Additional analysis of livestock data included the estimation of animal/subject seroprevalence by categorical variables, which included animal species (camel, cattle, goat, and sheep), animal sex (male and female), animal age (suckling/lamb/calf, weaner/youngling, and adult) and herd size (<200, 200-400,>400). For human data, seroprevalence was estimated for gender (male and female), age (4-20,21-40,41-60,>60), and the highest level of education completed (no education, primary, secondary, and above). Herd size and age for humans were considered as quantitative discontinuous variables. To determine the normality of our sample data, we initially performed the Shapiro-Wilk test to check for the normality of residuals. These two variables violated the linearity assumption and were transformed into both log-transformed and categorical variables and then compared with the outputs. We also determined the correlation between herd level exposure to *Brucella* spp. with

human exposure. We defined an exposed herd as any herd that had at least one seropositive animal.

To start the statistical analyses, we employed Chi-square (χ2) test to assess the independence of categorical variables. Additionally, we conducted univariable mixed-effects logistic regression models to investigate the association between independent variables and the outcome variable. We used the generalised linear mixed models to conduct both univariable and multivariable analyses. All variables with a p-value higher than 0.20 from the univariable analysis were selected for the multivariable analysis (37). The *lme4* package was used to fit the human and livestock data using the *glmer* function with random effects for household ID (37). The final model was done through backward elimination. Firstly, unconditional models without independent variables were fitted for the livestock and human data, and then global models for variables selected from the univariable data. We systematically eliminated variables with p values lower than 0.05 until we achieved the lowest Akaike's information criteria (AIC) in the multivariable model. The final models from this procedure were then assessed for the goodness of fit through inspection of residual plots against the fitted values from the model (38). Lastly, we tested for potential interaction effects within the selected variables by conducting a likelihood ratio test (LRT) was used to determine if there were potential interaction effects among product pairs of the factors in the final multivariable model (39). Furthermore, we estimated the ICC for within-herd and within-household clustering of animal and human brucellosis using the *icc* function available in the performance package (40)

Results

Descriptive statistics

A total of 2,157 animals (258 cattle, 953 goats, 849 sheep and 97 camels) were sampled from 237 households and tested for antibodies against *Brucella* spp. In five households (2.0%), animals were not available for sampling. In Garbatula, Sericho and Kinna wards, 977 (45.1%), 551 (25.5%) and 629 (29.2%) animals were sampled, respectively. Of the total sample, 1660 (77.0%) were female and 497 (23.0%) were males. The median herd size, combining cattle, sheep, goats, and camels was 176 (range: 8-1113), while the median herd/flock sizes for cattle, goats, sheep, and camels were 80 (range: 1–460), 80 (range: 8–546), 79 (range: 4-626) and 50 (range: 19-100), respectively. The median number of total animals sampled per household was 10 (range: 1–16).

This study also sampled 683 humans from 242 households. We achieved 97% of the minimum estimated sample (n = 704) due to consenting issues and smaller household sizes. Most humans were sampled in Garbatula ward (39.1%, n=267), followed by Sericho ward (34.8%, n=238) and Kinna ward 178 (26.1%). More males (83.0%, n=567) were sampled than females (17.0%, n=116). The median age of sampled subjects was 34 (range: 4-82), while the median number of individuals sampled in each household was 3 (range: 1–8).

Seroprevalence of *Brucella* spp., in livestock and humans

The overall animal and herd-level seroprevalence estimates of *Brucella* spp. in livestock were 10.4% (95 CI: 9.2 - 11.7) and 51.7% (95 CI: 47.9-55.7), respectively. The animal-level seroprevalence differed significantly between livestock species ($\chi 2 = 41.14$, $p < 0.01$). Camels had the highest seroprevalence of 19.6% (95 CI: 12.4-27.3), followed by cattle at 13.2 % (95 CI: 9.3-17.1), goats at 13.1% (95 CI: 11.1-15.3) and sheep 5.4% (95 CI: 4.0-6.9) in decreasing order (Table 1). Female animals had significantly higher seroprevalence ($\chi 2 = 11.94$, $p < 0.01$) of 11.6% (95 CI: 10.2-13.2) compared to male animals with a prevalence of 6.2% (95 CI: 4.4-8.4). When stratified by age, the seroprevalence increased significantly with age ($\chi 2 = 19.35$, $p < 0.01$) with adult animals having a higher seroprevalence of 11.9% (95 CI: 10.4-13.4) compared to young adults at 3.1% (95 CI: 1.4-4.8) and calves 2.8 % (95 CI: 0-5.7). The percentage of seropositive animals differed significantly by sampling locations ($\chi 2 = 16.29$, $p < 0.01$). Sericho had a higher seroprevalence estimate of 12.9% (95 CI: 10.3-15.7) compared to Kinna at 12.7% (95 CI: 10.3-15.4) and least in Garbatula at 7.5% (95 CI: 5.9-9.1).

The seroprevalence also differed significantly ($\chi 2 = 41.14$, $p < 0.01$) by the number of livestock species a farmer kept/owned. The results are given in Table 1.

Table 1. Univariable mixed-effects logistic regression analysis of the factors associated with livestock brucellosis seropositivity.

Variable	Category	n	Seroprevalence % (95% CI)	Odds Ratio (95% CI)	P-value
	Female	1660	11.6 (10.2-13.2)	1.0 (Ref.)	
Sex	Male	497	6.2 (4.4-8.4)	0.5 (0.3- 0.7)	0.001
	Goats	953	13.2 (9.3-17.1)	1.0 (Ref)	
	Cattle	258	13.1 (11.1-15.3)	0.9 (0.6-1.5)	0.020
	camel	97	19.6 (12.4-27.3)	1.8 (0.9-3.6)	0.020
Animal Species	Sheep	849	5.4 (4.0-6.9)	0.4 (0.2-0.5)	<0.001
	Calves/suckling	72	2. 8 (0.0-5.7)	1.0 (Ref.)	
	young adults	292	3.1 (1.4-4.8)	1.2 (0.2 - 5.8)	0.853
Animal age	Adults	1793	11.9 (10.4-13.4)	5.0 (1.2 - 21.5)	0.030
History of retained placenta	No	1368	12.8 (11.1-14.6)	1.0 (Ref.)	
	Yes	87	10.3 (5.8-17.2)	0.9 (0.4-2.2)	0.986
	No	1107	11.2 (9.5-13.1)	1.0 (Ref.)	
History of abortion	Yes	348	17.2 (13.5-21.2)	1.9 (1.3-2.8)	0.001
Introduction of animals into the herd	No	1614	11.5 (10.0-13.0)	1.0 (Ref.)	
	Yes	543	7.2 (5.2-9.2)	0.6 (0.4- 0.9)	0.024
Sharing grazing area with wildlife	No	302	11.3 (8.0-14.7)	1.0 (Ref.)	
	Yes	1855	10.2 (9.0-11.6)	0.8 (0.5-1.4)	0.534
	No	960	11.6 (9.7-13.6)	1.0 (Ref.)	
Breeding between herds	Yes	1197	9.4 (7.9-11.0)	0.8 (0.6-1.2)	0.306

Herd size	1-200	1247	9.1 (7.6-10.7)	1.0 (Ref.)	
	201-400	643	10.0 (7.8-12.2)	1.1 (0.7 - 1.7)	0.692
	>400	267	17.2 (13.1-21.9)	2.0 (1.1-3.4)	0.014
Sampling location	Kinna	629	12.7 (10.3-15.4)	1.0 (Ref.)	
	Garbatula	977	7.5 (5.9-9.1)	0.5 (0.3 - 0.8)	0.006
	Sericho	551	12.9 (-10.3-15.7)	1.0 (0.6 - 1.6)	0.922
CI confidence interval; Ref., reference category.					

The individual-level seroprevalence of *Brucella* spp. in humans was 54.0% (95 CI: 50.2-58.0) while the household seroprevalence was 86.4% (84.0 -89.0). Exposure to *Brucella* spp. was significantly higher ($\chi 2$ = 10.27.32, $p < 0.01$) in males 56.8% (95 CI: 52.7-61.2) compared to females 40.5% (95 CI: 31.9-49.9). When data were stratified by age, the individual human seroprevalence estimate increased with age, but the difference was not statistically significant. Seroprevalence estimates differed significantly by educational level ($\chi 2$ = 14.95.32, $p < 0.01$), with higher seroprevalence among those with no formal education at 57.5% (95 CI: 53.3-62.1) followed by those who completed primary education 47.0% (95 CI: 38.5-56.8) and it was least among those attained secondary or higher education 31.6% (95 CI: 21.7-47.6). The seropositivity of *Brucella* spp. in humans also differed significantly by sampling location ($\chi 2$ = 8.18, $p < 0.02$). Those sampled in Sericho had a higher seroprevalence 60.9% (95 CI: 54.6-67.2) compared to those in Garbatula 52.4% (95 CI: 46.4-58.8) and Kinna 47.2% (95 CI: 39.9-54.9). (Table 2)

Table 2. Univariable mixed-effects logistic regression analysis of the factors associated with human brucellosis seropositivity.

**Variable**	**Category**	**n**	**Seroprevalence % (95% CI)**	**Odds Ratio (95% CI)**	**P-value**
Gender	female	116	40.5 (31.9-49.9)	1 .0 (Ref.)	
	male	567	56.8 (52.7-61.2)	2.0 (1.3 - 3.1)	0.002
Age groups	≤20	155	47.7 (40-56.0)	1.0 (Ref.)	
	21-40	269	54.7 (48.7-60.9)	1.4 (0.9-2.1)	0.150
	41-60	177	60.5 (53.7-68.3)	1.7 (1.1-2.7)	0.021
	>60	82	50.0 (40.2-61.9)	1.2 (0.7-2.0)	0.627
Education level	Secondary and above	46	31.6 (21.7-47.6)	1.0 (Ref.)	
	Primary	117	47.0 (38.5-56.8)	1.9 (0.9- 4.0)	0.094
	No education	520	57.5 (53.3-62.1)	2.9 (1.5- 5.7)	0.002
Sampling locations	Kinna	178	47.2 (39.9-54.9)	1.0 (Ref.)	
	Garbatula	267	52.4 (46.4-58.8)	1.2 (0.8 -1.9)	0.302

	Sericho	238	60.9 (54.6-67.2)	1.8 (1.2-2.8)	0.009
Reported febrile illness in last 6 months	No	458	51.8 (47.2-56.6)	1.0 (Ref.)	
	Yes	225	58.7 (52.4-65.6)	1.4 (1.0-1.9)	0.075
Consumed milk (boiled or unboiled)	No	8	37.5 (12.5-70.9)		
	Yes	675	54.2 (50.4-58.2)	2.1 (0.5- 9.7)	0.336
Consumed unboiled milk (any of the species)	No	43	32.6 (20.9-47.8)	1.0 (Ref.)	
	Yes	640	55.5 (51.6-59.6)	2.6 (1.3 - 5.2)	0.007
Handled animals	No	23	13.0 (4.4-27.5)	1.0 (Ref.)	
	Yes	660	55.5 (51.7-59.5)	8.7 (2.5 -30.5)	0.001
Handled manure	No	385	55.3 (50.4-60.6)	1.0 (Ref.)	
	Yes	298	52.4 (46.6-58.3)	0.9 (0.6 -1.2)	0.464
Handled raw meat	No	165	48.5 (41.2-56.8)	1.0 (Ref.)	
	Yes	518	55.8 (51.5-60.4)	1.4 (0.9- 2.0)	0.112
Consumed bush meat.	No	531	52.9 (48.6-57.4)	1.0 (Ref.)	
	Yes	152	57.9 (50-65.9)	1.3 (0.8-1.9)	0.252
Herd exposure	Non-exposed	330	48.2 (42.7-53.9)	1.0(Ref)	
	Exposed	353	59.5 (54.4-64.9)	1.6 (1.2- 2.3)	0.005

CI confidence interval; Ref., reference category.

Results from univariable analyses done using livestock and humans' data.

The univariable mixed-effects logistic regression model results from the analysis of livestock data, with households as random effects, are shown in Table 1. On the specie level, camels and cattle had significantly higher odds of exposure to *Brucella* spp. when compared to goats. On the contrary, sheep had significantly lower odds of exposure than goats. We also found that female animals had higher odds of seropositivity compared to male animals. When considering animal age, only adult animals had significantly higher odds of seropositive compared to younger animals. We also observed higher odds of exposure in animals sampled from Garbatula Ward when compared to Kinna ward. Herds with more than 400 animals had higher odds of seropositivity compared to animals from smaller herds. Further analysis of risk factors associated with *Brucella* positivity showed that animals which experienced abortion had higher odds of seropositivity compared to animals that did not. Additionally, herds, where new animals were

introduced, reported higher odds of being seropositive compared to herds that did not introduce new animals. Finally, herds with at least two animal species had significantly higher odds of exposure to *Brucella* spp. compared to those with one species.

We did not find a significant association with *Brucella* spp. among animals from herds that shared grazing areas with wildlife, nor those where breeding within herds was practiced. These variables were excluded from the multivariable analysis.

Results for the human univariable models are shown in Table 2. The results show that male individuals had significantly higher odds of seropositivity compared to females. Similarly, those with no education had significantly higher odds of being seropositive than those who completed primary education or beyond. Geographically, people in Sericho ward had significantly higher odds of exposure to *Brucella* spp. than those in Kinna and Garbatula wards.

Among the risk factors for exposure to *Brucella* spp. analyzed, consuming unboiled from any species had higher odds of seropositivity than consuming boiled milk. Additionally, handling livestock was identified as a significant risk factor, but we found no significant association with *Brucella* spp. exposure among those who handled wild animals or consumed bushmeat.

Multivariable analyses

The results from the multivariable mixed-effects model for animal data are summarized in Table 3. Four fixed effects were significant predictors of *Brucella* spp. exposure among animals, including animal sex, age, species, and herd size. Specifically, female animals had 1.7 times higher odds of being seropositive compared to male animals. The odds of being seropositive increased with increasing animal age, and adult animals were seven times more likely to be exposed to *Brucell*a spp. than calves. Sheep had significantly lower odds of being seropositive compared to goats. However, cattle or camel did not have a significant effect on the likelihood of exposure. Animals in larger herd sizes (above 400 animals) had significantly higher odds of *Brucella* spp. exposure compared to animals in smaller herds, with the likelihood of exposure increasing with the increase in the size of the herds. The history of abortion variable in sampled animals was excluded from the multivariable models because it only affected adult females and would not improve the model's predictability, despite its significant association with exposure to brucellosis in the univariable analysis. The ICC estimate for the household random effect in the final model was 0.138 (95% CI: 0.554-1.046), while the LRT $\chi 2$ for the product pairs created for the independent variables in the final models was not significant ($p \leq 0.05$), suggesting that the model's predictors were not significantly collinear.

Table 3. Multivariable mixed-effects logistic regression analysis of the factors associated with livestock brucellosis seropositivity.

Variable	Category	Odds Ratio (95% CI)	SE	Z	P- value
**Fixed effects**					
**Sex**	Male	1.0 (Ref.)			
	Female	1.6 (1.1-2.5)	0.217	2.195	0.028
**Age category**	Calves	1.0 (Ref.)			
	Weaners	1.6 (0.2-8.2)	0.827	0.591	0.554
	Adults	7.6 (1.7-33.8)	0.763	2.655	0.008
**Animal species**	Goats	1.0 (Ref.)			
	Cattle	1.3 (0.8-2.1)	0.254	0.992	0.321
	Camel	2.4 (1.2-4.8)	0.355	2.470	0.140
	Sheep	0.3 (0.2-0.5)	0.189	-5.587	<0.001
**Herd size**	1-200	1.0 (Ref.)			
	201-400	1.2 (0.7-1.8)	0.220	0.644	0.520
	Above 400	2.0 (1.2- 3.5)	0.275	2.611	0.009

Ref., reference category; CI, confidence interval; SE, standard error.
LRT-Likelihood-Ratio Test
Log likelihood =-655.55, number of observations =2157, number of households= 237
The variance for Household ID as the random effect variable was 0.625 (95% CI: 0.5537 - 1.046), std dev=0.79

The outputs from the multivariable mixed-effects model for human data are summarized in Table 4. Three variables were significant: gender, education level, and herd exposure. Males had twice the odds of exposure compared to females, while individuals who did not complete any formal education had three times higher odds of exposure compared to those who completed secondary education or higher. The odds of exposure decreased with an increase in educational attainment.

Consumption of unboiled milk did not significantly affect the likelihood of exposure. There was a strong correlation between human seropositivity and livestock seropositivity for Brucella, with almost two-fold higher odds of exposure to people from households with at least one seropositive animal than those with not. The ICC value calculated from the variance estimates of the final multivariable model was 0.03 (95% CI; <0.01 - 0.74).

Table 4. Multivariable mixed-effects logistic regression analysis of the factors associated with human brucellosis seropositivity.

Variable	Category	Odds Ratio (95% CI	SE	Z	P-value
**Fixed effects**					
**Gender**	Female	1 (Ref)			
	Male	2.0 (1.3- 3.1)	0.224	3.042	0.002
**Handling animals**	No	1 (Ref)			
	Yes	6.2 (1.7-22.3)	0.172	2.813	0.005
**Highest Education level completed**	Secondary and above	1 (Ref)			
	Primary	1.8 (0.9-3.9)	0.385	1.574	0.103
	No education	2.9 (1.5- 5.8)	0.347	3.092	0.002
**Herd exposure**	No	1 (Ref)			
	Yes	1.4 (1.0-2.0)	0.171	2.127	0.033
**Location**	Kinna	1 (Ref)			
	Garbatulla	1.3(0.9-2.0)	0.213	1.356	0.175
	Sericho	1.6(1.1-2.5)	0.220	2.252	0.024

Ref., reference category; CI, confidence interval; SE, standard error.
Log likelihood =-445.5, number of observations =683, number of households= 242
The variance for Household ID as the random effect variable was 0.126 (95% CI: 0.00- 0.744), SE 0.764

Discussion

This linked study aimed to estimate the seroprevalence of *Brucella* spp. among humans and animals from the same household and to determine the risk factors associated with *Brucella* spp. seropositivity in a pastoralist area in northern Kenya.

The study found a high level of brucellosis seroprevalence at the individual and household levels, with individual seroprevalence of 54% (95%CI: 50.2-58.0) and household seroprevalence of 86.4% (95% CI: 84.0 -89.0). The individual seroprevalence of 54% is higher than the 14-46.5% range reported in comparable studies conducted among pastoralist communities (22,23,25,41). These high seroprevalence estimates could be due to the study participants' constant exposure to infected animals and animal products. Additionally, the study sampled more men and older individuals (mean age of 34), compared to other studies (22,23), and men are more prone to infection compared to females due to occupational exposure, while older individuals have had a longer exposure time to the disease (42). The high seroprevalence highlights the high endemicity of the disease in our study area due to the constant exposure of study participants to infected animals and animal products.

We observed high animal- and herd-level seroprevalence estimates of *Brucella* spp. in livestock. The observed overall seroprevalence was lower than that reported in seroprevalence studies in Marsabit and Baringo counties in Kenya (22,24,25) but higher than reported in Garissa County in

Kenya (23) and the Somali region of Ethiopia (41). The seroprevalence varied by species, with camels having the highest seroprevalence, followed closely by goats and cattle, while sheep had the lowest seroprevalence. The high seroprevalence in camels has also been observed in studies conducted in Isiolo County (43,44) , while studies in neighbouring Marsabit County report lower estimates (22,25). Although camels are not the primary hosts for *Brucella* spp., the high seroprevalence detected in this study suggests their potential role in maintaining brucellosis transmission in the area. The Borana community in Isiolo County traditionally keeps cattle, goats, and sheep but is increasingly embracing camels due to their resilience to climate variability (45,46). The lower seroprevalence estimates in sheep have been reported in other studies (23,43), which utilised similar testing strategies, but there is currently limited evidence on why sheep have lower *Brucella* exposure levels compared to other ruminant species such as goats and cattle.

The study utilized multivariable models to identify factors associated with *Brucella* spp. seropositivity in livestock. Female animals had almost two-fold higher odds of exposure than males. One main reason for this high likelihood is increased brucellosis transmission through reproductive fluids. Female animals can be exposed to bacteria during breeding, pregnancy, and birth, making them more vulnerable to infection (47). Additionally, female animals are kept longer for reproduction and milk production, which is a common practice in pastoralist communities (22,23,25). In contrast, male animals are typically sold to meet the household's needs, such as food, healthcare, veterinary services, and education.

Adult animals were found to be five times more likely to be exposed to brucellosis compared to calves. Older animals have greater exposure time, travel further and therefore have more opportunities to mix with other herds, leading to increased disease transmission. Similar findings have been reported in Marsabit County and Garissa County in Kenya (22,23). Further, the study found that female animals that had experienced abortion in the previous year were more likely to be seropositive for brucellosis at the univariable analysis. However, this factor was not included in the multivariable analysis, as it affected only adult female animals. Nonetheless, this finding emphasizes the well-known association between spontaneous abortion in animals and brucellosis infection(48,49). Despite the established link between brucellosis and spontaneous abortion in livestock, pastoralists may not always associate spontaneous abortions in their animals with brucellosis (50). As a result, they may continuously expose themselves to the disease while handling infected abortus materials.

Brucellosis prevalence was highly correlated with herd size, with larger herds having a significantly higher risk of exposure to the disease. Large herds are often associated with poor sanitation, clustering of animals, and mixing of animals from different herds and species. Several studies in Africa have found a similar correlation between brucellosis seropositivity and herd size (23,49,51).

The multivariable model on humans found several factors to be associated with brucellosis at the individual level. These included being male and having a lower than secondary education to be

significant in the analysis, and this may be because the sample included older individuals with a median age of 34, or its effect may have been diluted or masked by other factors in the sample. Males were found to have a higher seroprevalence than women, with a seropositivity rate of 57% compared to 41% in females. Men had two-fold higher odds of exposure compared to women. Worldwide, brucellosis is more frequently detected in males than females. This difference in odds of infection between the genders can be attributed to occupational exposure, such as frequent contact with infected animals or contaminated animal products while herding, milking, slaughtering, or assisting with animal birth(42). Similar findings have been documented in local studies (22,23). Additionally, we found that individuals who regularly handled animals (participated in herding, milking, slaughtering, or assisting in animal births) had six times higher odds of being seropositive than those who did not. These findings highlight the importance of proper hygiene practices when working with animals or animal products and the need for education and awareness campaigns to reduce the risk of brucellosis transmission in high-risk populations, such as male herders.

The consumption of milk and milk products is a crucial aspect of the diet of Borana pastoralists, but it can also pose serious health risks, particularly when consumed raw. Despite the risks, a vast majority (94%) of our study participants reported consuming unboiled milk in the three months preceding the study. However, regular consumption of unboiled milk was not a significant risk factor in the multivariable analysis. We believe this is because almost all participants reported they consume raw milk. Unlike our study, pastoralist communities in Isiolo and Marsabit County have also strongly linked the consumption of raw milk to brucellosis exposure (22,52). Boiling milk is a simple and effective way to kill milk-borne pathogens including *Brucella* spp. while preserving the nutritional value of the milk. However, a previous study in southern Ethiopia found that Borana pastoralists considered boiling milk to reduce nutrient value and make the milk lack taste (53), emphasizing the need to find a balance between preserving cultural practices and minimizing health risks.

The poor literacy levels in the study area were reflected in the fact that most participants had not received any formal education. Attaining secondary education or higher was found to be protective against exposure to *Brucella* spp. and those who did not complete the lowest level of formal education had about three times higher odds of exposure to brucellosis. This finding aligns with previous observations by a study conducted in neighboring Marsabit county (22) , which is also inhabited by the *Borana* community. These results have important implications for public health interventions aimed at creating awareness about brucellosis. Higher education levels are associated with better awareness of the disease (54,55), and therefore designing culturally sensitive, simple, and accessible public education measures would be critical to reducing disease transmission among less educated pastoralists.

Human exposure to brucellosis had a 1.5 times higher likelihood of occurring within households that had a positive herd. This shows that many human infections are transmitted through close contact with the household's infected animal materials. This finding is consistent with other

studies conducted in neighbouring areas (22,23). However, the correlation between human and animal *Brucella* spp. positivity was much less in this study, suggesting that the risk of transmission at the household level was lower in our study area. This is supported by the low intra-class correlation (ICC) of 0.03 in humans, which indicates that human exposure to *Brucella* spp. was not correlated among individuals within households. This is not surprising because human-to-human transmission of brucellosis is rare, and most infections are typically transmitted through the consumption of contaminated animal products or contact with infected animals.

Finally, our data reveal that individuals living in Sericho ward have significantly higher *Brucella* spp. seropositivity rates compared to those in Kinna and Garbatula Wards. Sericho is a remote, low-lying area nomadic pastoralists prefer for its flat terrain and extensive grazing lands. However, unlike Kinna and Garbatula, where many pastoralists live in settlements with some amenities such as schools and health facilities, Sericho is quite underserved, and most people have limited access to healthcare and education (30), which may contribute to higher seropositivity rates. Additional research is necessary to understand better the reasons behind the higher seropositivity rates in Sericho ward.

Limitations

Our study had a few limitations. Due to insecurity, we could not access a section of the study area, which could have biased our estimates and affected the generalizability of the study. The study was conducted during a hot and dry season, a period often characterized by adaptation strategies, including an increase in droughts-adapting livestock, herd splitting, household splitting and clustering of herds for security reasons and in areas proximal to pasture and water for their livestock. (56,57)

Conclusion and recommendations

Our study reports a high prevalence of brucellosis in humans and livestock in the study area, underscoring the need for targeted prevention and control measures to curb its spread. The study revealed several factors associated with *Brucella* spp. seropositivity in humans and livestock, including animal age, herd size, livestock species, gender, education level, and herd exposure. Enhancing animal health management procedures, encouraging appropriate hygiene standards when handling animals, and promoting the consumption of safe animal products are three vital ways of mitigating disease transmission. This can involve creating more public awareness about the disease to minimize the risk of disease transmission and increasing veterinary outreach services by the County Government of Isiolo to pastoralists, such as vaccination. In addition, the One Health approach applied in this study emphasizes the significance of understanding the interactions between humans and animals in transmitting and controlling zoonotic diseases, showing the need to establish an integrated One Health surveillance system in such high-risk populations. Future research should examine the efficacy of

measures to reduce these factors to mitigate the spread of brucellosis and the socio-economic impacts of brucellosis in the region.

Acknowledgements

We thank all people of Garbatula subcounty for accepting to join the study. We also thank the public and animal health officials from Isiolo County for approving the study to be implemented in their area. Additionally, we thank the human and animal health personnel who assisted in sampling study participants. This study was implemented under the project 'Co-infection with Rift Valley fever virus, *Brucella* spp. and *Coxiella burnetii* in humans and animals in Kenya: Disease burden and ecological factors' funded by Defense Threat Reduction Agency, Grant No. HDTRA11910031. The content of the information does not necessarily reflect the position or the policy of the federal government, and no official endorsement should be inferred. We also acknowledge the CGIAR Fund Donors (https://www.cgiar.org/funders).

References

1. Franc KA, Krecek RC, Häsler BN, Arenas-Gamboa AM. Brucellosis remains a neglected disease in the developing world: A call for interdisciplinary action. BMC Public Health [Internet]. 2018 Jan 11 [cited 2022 Nov 30];18(1):1–9. Available from: https://bmcpublichealth.biomedcentral.com/articles/10.1186/s12889-017-5016-y

2. de Figueiredo P, Ficht TA, Rice-Ficht A, Rossetti CA, Adams LG. Pathogenesis and Immunobiology of Brucellosis: Review of Brucella–Host Interactions. Am J Pathol [Internet]. 2015;185(6):1505–17. Available from: http://www.sciencedirect.com/science/article/pii/S0002944015001832

3. Doganay M, Aygen B. Human brucellosis: an overview. International Journal of Infectious Diseases [Internet]. 2003;7(3):173–82. Available from: http://www.sciencedirect.com/science/article/pii/S120197120390049X

4. Young EJ. An Overview of Human Brucellosis. Clinical Infectious Diseases [Internet]. 1995 Aug 1;21(2):283–90. Available from: https://doi.org/10.1093/clinids/21.2.283

5. Godfroid J, Scholz HC, Barbier T, Nicolas C, Wattiau P, Fretin D, et al. Brucellosis at the animal/ecosystem/human interface at the beginning of the 21st century. Prev Vet Med. 2011 Nov 1;102(2):118–31.

6. Corbel MJ. Brucellosis in humans and animals [Internet]. Geneva PP - Geneva: World Health Organization; 2006. Available from: https://apps.who.int/iris/handle/10665/43597

7. Köse Ş, Senger SS, Akkoçlu G, Kuzucu L, Ulu Y, Ersan G, et al. Clinical manifestations, complications, and treatment of brucellosis: Evaluation of 72 cases. Turk J Med Sci. 2014;44(2):220–3.

8. Buzgan T, Kasim M, Irmak H, Irfan A, Karsen H, Evirgen O, et al. International Journal of Infectious Diseases Clinical manifestations and complications in 1028 cases of brucellosis : a retrospective evaluation and review of the literature. International Journal of Infectious Diseases [Internet]. 2010;14(6):e469–78. Available from: http://dx.doi.org/10.1016/j.ijid.2009.06.031

9. Goonaratna C. Brucellosis in Humans and Animals. Ceylon Medical Journal. 2009;52(2):66.

10. Tuon FF, Gondolfo RB, Cerchiari N. Human-to-human transmission of Brucella – a systematic review. 2017;22(5):539–46.

11. Salata RA. Brucellosis. In: Goldman's Cecil Medicine: Twenty Fourth Edition. Elsevier Inc.; 2011. p. 1891–3.

12. Dean AS, Crump L, Greter H, Schelling E, Zinsstag J. Global Burden of Human Brucellosis: A Systematic Review of Disease Frequency. PLoS Negl Trop Dis. 2012;6(10).

13. Andriopoulos P, Tsironi M, Deftereos S, Aessopos A, Assimakopoulos G. Acute brucellosis : presentation , diagnosis , and treatment of 144 cases. 2007;52–7.

14. Madkour MM M. Madkour's Brucellosis. New York: Springer Verlag; 2001.

15. Cárdenas L, Peña M, Melo O, Casal J. Risk factors for new bovine brucellosis infections in Colombian herds. BMC Vet Res [Internet]. 2019 Mar 7 [cited 2021 Mar 23];15(1):81. Available from: https://bmcvetres.biomedcentral.com/articles/10.1186/s12917-019-1825-9

16. Ducrotoy MJ, Bertu WJ, Ocholi RA, Gusi AM, Bryssinckx W, Welburn S, et al. Brucellosis as an Emerging Threat in Developing Economies: Lessons from Nigeria. Crump JA, editor. PLoS Negl Trop Dis [Internet]. 2014 Jul 24 [cited 2021 Mar 23];8(7):e3008. Available from: https://dx.plos.org/10.1371/journal.pntd.0003008

17. Neta AVC, Mol JPS, Xavier MN, Paixão TA, Lage AP, Santos RL. Pathogenesis of bovine brucellosis [Internet]. Vol. 184, Veterinary Journal. Vet J; 2010 [cited 2021 Mar 23]. p. 146–55. Available from: https://pubmed.ncbi.nlm.nih.gov/19733101/

18. Xavier MN, Paixão TA, Poester FP, Lage AP, Santos RL. Pathological, Immunohistochemical and Bacteriological Study of Tissues and Milk of Cows and Fetuses Experimentally Infected with Brucella abortus. J Comp

Pathol [Internet]. 2009 Feb [cited 2021 Mar 23];140(2–3):149–57. Available from: https://pubmed.ncbi.nlm.nih.gov/19111839/

19. Goodwin ZI, Pascual DW. Brucellosis vaccines for livestock. Vet Immunol Immunopathol [Internet]. 2016 Nov 15 [cited 2021 Mar 23];181:51–8. Available from: https://pubmed.ncbi.nlm.nih.gov/27032465/

20. Godfroid J, al Dahouk S, Pappas G, Roth F, Matope G, Muma J, et al. A "One Health" surveillance and control of brucellosis in developing countries: Moving away from improvisation. Comp Immunol Microbiol Infect Dis [Internet]. 2013;36(3):241–8. Available from: http://www.sciencedirect.com/science/article/pii/S0147957112001014

21. Munyua P, Bitek A, Osoro E, Pieracci EG, Muema J, Mwatondo A, et al. Prioritization of Zoonotic Diseases in Kenya, 2015. PLoS One [Internet]. 2016 Aug 1 [cited 2023 Mar 24];11(8):e0161576. Available from: https://journals.plos.org/plosone/article?id=10.1371/journal.pone.0161576

22. Osoro EM, Munyua P, Omulo S, Ogola E, Ade F, Mbatha P, et al. Strong association between human and animal brucella seropositivity in a linked study in Kenya, 2012-2013. American Journal of Tropical Medicine and Hygiene. 2015 Aug 1;93(2):224–31.

23. Kairu-Wanyoike S, Nyamwaya D, Wainaina M, Lindahl J, Ontiri E, Bukachi S, et al. Positive association between Brucella spp. seroprevalences in livestock and humans from a cross-sectional study in Garissa and Tana River Counties, Kenya. PLoS Negl Trop Dis [Internet]. 2019 [cited 2023 Apr 5];13(10):e0007506. Available from: https://journals.plos.org/plosntds/article?id=10.1371/journal.pntd.0007506

24. Lokamar PN, Kutwah MA, Munde EO, Oloo D, Atieli H, Gumo S, et al. Prevalence of brucellosis in livestock keepers and domestic ruminants in Baringo County, Kenya. PLOS Global Public Health [Internet]. 2022 Aug 15 [cited 2023 Apr 5];2(8):e0000682. Available from: https://journals.plos.org/globalpublichealth/article?id=10.1371/journal.pgph.0000682

25. Muema J, Oboge H, Mutono N, Makori A, Oyugi J, Bukania Z, et al. Sero – epidemiology of brucellosis in people and their livestock: A linked human – animal cross-sectional study in a pastoralist community in Kenya. Front Vet Sci. 2022 Nov 18;9:1816.

26. Njeru J, Wareth G, Melzer F, Henning K, Pletz MW, Heller R, et al. Systematic review of brucellosis in Kenya: disease frequency in humans and animals and risk factors for human infection. BMC Public Health 2016 16:1 [Internet]. 2016 Aug 22 [cited 2023 Mar 12];16(1):1–15. Available from:

https://bmcpublichealth.biomedcentral.com/articles/10.1186/s12889-016-3532-9

27. Godfroid J, al Dahouk S, Pappas G, Roth F, Matope G, Muma J, et al. A one health surveillance and control of brucellosis in developing countries: moving away from improvisation. Comp Immunol Microbiol Infect Dis. 2013 May;36(3):241–8.

28. Godfroid J. Brucellosis in livestock and wildlife: Zoonotic diseases without pandemic potential in need of innovative one health approaches. Archives of Public Health [Internet]. 2017 Sep 11 [cited 2022 Nov 30];75(1):1–6. Available from: https://link.springer.com/articles/10.1186/s13690-017-0207-7

29. Zinsstag J, Schelling E, Roth F, Bonfoh B, de Savigny D, Tanner M. Human benefits of animal interventions for zoonosis control. Emerg Infect Dis [Internet]. 2007 [cited 2021 Mar 15];13(4):527–31. Available from: https://pubmed.ncbi.nlm.nih.gov/17553265/

30. Isiolo County Integrated Development Plan 2018-2022. 2018 [cited 2023 Mar 24]; Available from: https://repository.kippra.or.ke/handle/123456789/1409

31. 2019 Kenya Population and Housing Census Volume I: Population by County and Sub-County - Kenya National Bureau of Statistics [Internet]. [cited 2023 Mar 12]. Available from: https://www.knbs.or.ke/?wpdmpro=2019-kenya-population-and-housing-census-volume-i-population-by-county-and-sub-county

32. Wanyoike F, Njiru N, Kutu A, Chuchu S, Wamwere-Njoroge G, Mtimet N. Kenya Accelerated Value Chain Development Program (AVCD)-livestock component Analysis of livestock and fodder value chains in arid and semi-arid lands in Kenya. 2018;

33. Fosgate GT. Practical sample size calculations for surveillance and diagnostic investigations. Journal of Veterinary Diagnostic Investigation [Internet]. 2009 Jan 1 [cited 2023 Mar 11];21(1):3–14. Available from: https://journals.sagepub.com/doi/full/10.1177/104063870902100102

34. R Core Team (2019) R A Language and Environment for Statistical Computing. R Foundation for Statistical Computing, Vienna, Austria. - References - Scientific Research Publishing [Internet]. [cited 2022 Jun 28]. Available from: https://www.scirp.org/(S(lz5mqp453edsnp55rrgjct55))/reference/ReferencesPapers.aspx?ReferenceID=2631126

35. Warnes: gmodels: Various R programming tools for... - Google Scholar [Internet]. [cited 2022 Dec 1]. Available from:

https://scholar.google.com/scholar?cluster=8652668821377558 38&hl=en&as_sdt=2005&sciodt=0,5&scioq=Package+%E2%80%98gmodels%E2%80%99+Warnes+2015

36. epiR (Stevenson, 2013) - Google Scholar [Internet]. [cited 2022 Dec 1]. Available from: https://scholar.google.com/scholar?hl=en&as_sdt=0%2C5&scioq=Package+%E2%80%98gmodels%E2%80%99+Warnes+2015&q=epiR+%28Stevenson%2C+2013%29+&btnG=

37. Bates D, Mächler M, Bolker B, arXiv:1406.5823 SW arXiv preprint, 2014 undefined. Fitting linear mixed-effects models using lme4. arxiv.org [Internet]. [cited 2022 Dec 1]; Available from: https://arxiv.org/abs/1406.5823

38. Harrison XA, Donaldson L, Correa-Cano ME, Evans J, Fisher DN, Goodwin CED, et al. A brief introduction to mixed effects modelling and multi-model inference in ecology. PeerJ [Internet]. 2018 May 23 [cited 2022 Dec 1];2018(5):e4794. Available from: https://peerj.com/articles/4794

39. Dohoo IR, Ducrot C, Fourichon C, Donald A, Hurnik D. An overview of techniques for dealing with large numbers of independent variables in epidemiologic studies. Prev Vet Med [Internet]. 1997 [cited 2022 Dec 1];29(3):221–39. Available from: https://pubmed.ncbi.nlm.nih.gov/9234406/

40. Lüdecke D, Ben-Shachar MS, Patil I, Waggoner P, Makowski D. performance: An R Package for Assessment, Comparison and Testing of Statistical Models. [cited 2023 Mar 11]; Available from: https://doi.org/10.21105/joss.03139

41. Ibrahim M, Schelling E, Zinsstag J, Hattendorf J, Andargie E, Tschopp R. Sero-prevalence of brucellosis, Q-fever and Rift Valley fever in humans and livestock in Somali Region, Ethiopia. PLoS Negl Trop Dis [Internet]. 2021 [cited 2023 Apr 5];15(1):e0008100. Available from: https://journals.plos.org/plosntds/article?id=10.1371/journal.pntd.0008100

42. Doganay M, Aygen B. Human brucellosis: an overview. International Journal of Infectious Diseases. 2003 Sep 1;7(3):173–82.

43. Njeru J, Nthiwa D, Akoko J, Oyas H, Bett B. Incidence of Brucella infection in various livestock species raised under the pastoral production system in Isiolo County, Kenya. BMC Vet Res [Internet]. 2021 Dec 1 [cited 2023 Mar 11];17(1):1–12. Available from: https://bmcvetres.biomedcentral.com/articles/10.1186/s12917-021-03036-z

44. Muturi M, Akoko J, Nthiwa D, Chege B, Nyamota R, Mutiiria M, et al. Serological evidence of single and mixed infections of Rift Valley fever virus, Brucella spp. and Coxiella burnetii in dromedary camels in Kenya. PLoS Negl Trop Dis [Internet]. 2021 Mar 1 [cited 2023 Mar 15];15(3):e0009275. Available from: https://journals.plos.org/plosntds/article?id=10.1371/journal.pntd.0009275

45. Degen AA. Transformation of Borana from nomadic pastoralists to agropastoralists and shift of livestock from cattle to include more goats, camels and sheep in Southern Ethiopia. International Journal of Business and Globalisation. 2011;6(3–4):292–312.

46. Kagunyu AW, Wanjohi J. Camel rearing replacing cattle production among the Borana community in Isiolo County of Northern Kenya, as climate variability bites. Pastoralism [Internet]. 2014 Dec 1 [cited 2023 Feb 28];4(1):1–5. Available from: https://pastoralismjournal.springeropen.com/articles/10.1186/s13570-014-0013-6

47. OIE. (2019). Brucellosis (Brucella abortus, B. melitensis... - Google Scholar [Internet]. [cited 2023 Mar 15]. Available from: https://scholar.google.com/scholar?hl=en&as_sdt=0%2C5&q=OIE.+%282019%29.+Brucellosis+%28Brucella+abortus%2C+B.+melitensis+and+B.+suis%29+%28Infection+with+B.+ovis%29.+In%3A+Manual+of+Diagnostic+Tests+and+Vaccines+for+Terrestrial+Animals.&btnG=

48. Sing A. Zoonoses—infections affecting humans and animals: Focus on public health aspects. Zoonoses-Infections Affecting Humans and Animals: Focus on Public Health Aspects. 2015 Jan 1;1–1143.

49. Ndazigaruye G, Mushonga B, Kandiwa E, Samkange A, Segwagwe BE. Prevalence and risk factors for brucellosis seropositivity in cattle in Nyagatare District, Eastern Province, Rwanda. J S Afr Vet Assoc [Internet]. 2018 Dec 5 [cited 2023 Feb 27];89. Available from: /pmc/articles/PMC6295791/

50. Mburu CM, Bukachi SA, Tokpa KH, Fokou G, Shilabukha K, Ezekiel M, et al. Lay attitudes and misconceptions and their implications for the control of brucellosis in an agro-pastoral community in Kilombero district, Tanzania. PLoS Negl Trop Dis [Internet]. 2021 Jun 1 [cited 2023 Feb 28];15(6):e0009500. Available from: https://journals.plos.org/plosntds/article?id=10.1371/journal.pntd.0009500

51. Terefe Y, Girma S, Mekonnen N, Asrade B. Brucellosis and associated risk factors in dairy cattle of eastern Ethiopia. Trop Anim Health Prod [Internet].

2017 Mar 1 [cited 2023 Feb 27];49(3):599–606. Available from: https://pubmed.ncbi.nlm.nih.gov/28176187/

52. Onyango DLA, Guitian J, Musallam I. Brucellosis risk factors and milk hygiene handling practices in pastoral communities in Isiolo county, Kenya. Vet Med Sci [Internet]. 2021 Jul 1 [cited 2022 Nov 17];7(4):1254–62. Available from: https://onlinelibrary.wiley.com/doi/full/10.1002/vms3.453

53. Amenu K, Wieland B, Szonyi B, Grace D. Milk handling practices and consumption behavior among Borana pastoralists in southern Ethiopia. J Health Popul Nutr [Internet]. 2019 Feb 7 [cited 2022 Nov 17];38(1):6. Available from: https://jhpn.biomedcentral.com/articles/10.1186/s41043-019-0163-7

54. Alqahtani YA, Shati AA, Al-Qahtani SM, Asseri AA, Alhanshani AA, Alqahtani FM, et al. Knowledge, Attitudes, and Practices Regarding Brucellosis among Parents in Aseer Region, Southwestern Saudi Arabia. Healthcare 2021, Vol 9, Page 1541 [Internet]. 2021 Nov 11 [cited 2023 Feb 27];9(11):1541. Available from: https://www.mdpi.com/2227-9032/9/11/1541/htm

55. Mufinda FC, Boinas F, Nunes C. Prevalence and factors associated with human brucellosis in livestock professionals. Rev Saude Publica [Internet]. 2017 [cited 2023 Feb 28];51. Available from: https://pubmed.ncbi.nlm.nih.gov/28658364/

56. Lelamo LL, Shenkut BT, Abdilahi AH. Drought characteristics and pastoralists' response strategies in Korahey zone, Somali regional state, Eastern Ethiopia. Sci Afr. 2022 Jul 1;16:e01254.

57. Major Climate Change Adaptation and Coping Strategies in Borana, Southern Ethiopia: A Review. Journal of Resources Development and Management. 2020 Oct;
