# Supplementary material for: Seroprevalence and related risk factors of Brucella spp. in livestock and humans in Garbatula subcounty, Isiolo county, Kenya
Source: PLoS Negl Trop Dis. 2023 Oct 16;17(10):e0011682. doi: 10.1371/journal.pntd.0011682 (PMC10602376; doi:10.1371/journal.pntd.0011682)
Supplement: Attachment — Submitted filename: Reviewed Seroprevalence of Brucella spp.docx [file pntd.0011682.s004.docx]

**PLOS Neglected Tropical Diseases**

Seroprevalence of *Brucella* spp. in livestock and humans in Isiolo county in Kenya

**Type:** Research Article

**Manuscript Number:** PNTD-D-23-00564

**The Editor**

**PLOS Neglected Tropical Diseases**

This is an important record of brucellosis occurrence in Kenya, and it provides vital information about the sero-epidemiology of the disease in the selected region of the country. Furthermore, this also has implications for other parts of Africa.

**Comments**

Can the authors give reasons why Rose Bengal test (RBT) was not used for screening?, or for adaptation of brucellosis testing in series which was not performed.

How would the authors explain the elimination of false positives because of the widely used serologic ELISAs for the detection of brucellosis which is very high, to the extent that some laboratories apply not only the CFT, but parallel with it, the classic Rose Bengal (RBT) or/and serum agglutination (SAT) tests for confirmation.

There is the possibility of cross-reaction of Gram-negative bacterium species or other unknown organisms or molecular antigens that are generally considered as contributing in false positives using ELISA, how would the authors clarify this in this study.

Serology alone can only be a presumptive test since other pathogens (for example, Y.

337 enterocolitica, O:9) can cross-react with the tests, thereby leading to false-positive results.

Can the authors elaborate more on the vaccination status or strategies in the study areas, as seropositivity could have been due to reaction to vaccination.

- **Abstract**
- Page 2, line 31: Remove “high” and replace with “an”
- Page 2, line 36: insert “anti-brucella” before antibody detection
- Page 2, line 44: change “*spp*” in italics to “spp.”
- Page 2, line 45: put :”the” before “disease spread”
- Page 2, line 51: Replace “Brucella” with “*Brucella*” in italics
- Page 2, line 63: Put a space between “globally” and “(1,2).”
- Page 3, line 69: Put a space between “species” and (6)
- Page 3, line 69: Replace “consuming” with “consumption of”
- Page 3, line 87: Put space between “complications” and “(14)”
- Page 3, line 88: Remove “-“ between “water-contaminated”
- Page 3, line 89: Replace “like” with “such as”
- Page 3, line 94: Insert coma “,” after developed countries and insert “it” before “is”
- Page 3, line 95: Remove full stop “.” after “Middle East”
- Page 3, line 97: Put space between “numbers” and “(20)”
- Page 3, lines 102 – 103: the authors may wish to use “%” in place of “per cent”
- Page 3, line 103: Insert space between “livestock” and “(22 – 26)”
- Page 3, line 105: Replace “one-health” with “One-Health”
- Page 3, line 105: Replace “includingntegrated” with “including intergrated”
- Page 3, line 108: insert “.” After “spp”
- **Materials and methods**
- Page 4, line 116: Remove “sought and”
- Page 4, line 129, 134 and 135: Insert space between “459m”, “kilometre(31) and “inhabitants(31)”
- Page 5, line 148: Insert space between “size(n)”
- Page 6, line 176: Replace “these” with “them”
- Page 6, line 181 – 182: Can the authors using a uniform sign for degrees (°) ?? See 4-8°C differs from -20 ^0^C
- Page 6, line 184: Insert degree “°” between “20” and “C”
- Page 7, line 211: Replace “Lastly” with “Finally”
- Page 7, line 218: Remove “against” and insert space between “120” and “%”
- Page 8: Would the authors give the reference sensitivity and specificity for the serological tests used?
- Page 8, line 238: Insert “C” after “37°) and insert space between “(pNPP)” and “substrate”
- Page 8, line 240: 37°C for 30 ???? what?
- Page 8, line 241: Replace “done” with “conducted”
- Page 8,line 249: Remove “)” after “OD”
- Page 8,lines 267 and 268: reduce spacing and insert appropriate spacing between numbers in brackets
- Page 8, line 289: Replace “Lastly” with “Finally”
- Page 8, line 290: Insert “, this” before “was”
- **Results**
- Page 9, line 299: Insert “anti-*Brucella”* before “antibodies”
- Page 10, line 312: Remove “,” after “spp.”
- Page 11, line 334: Insert “95% CI:” before “84.0-89.0”
- Page 11, line 340: insert “who” after “those”
- Page 11, line 344: Romove “.” After “(95% CI: 39.9-54.9)” and insert “.” After (Table).
- Page 13, line 371: Replace “unboiled” with “raw milk”
- Page 13, line 372: Replace “boiled” with “pasteurized”
- Page 14, line 403: Replace “unboiled” with “raw milk”
- Page 14, line 404: Replace “Brucella” with “*Brucella*” in italics
- Page 14, line 406: Replace “with not” with “without”
- Page 14, line 407: Remove “<” before “0.01”
- Page 15, line 414: Replace “risk factors associated” with “associated risk factors”
- Page 15, lines 424 -426: Repetition “The high seroprevalence highlights the high endemicity of the disease in our study area due to the constant exposure of study participants to infected animals and animal products.”
- Page 16, line 434: Insert “,” after “Although”
- Page 16, line 458: Insert space between “infection” and “(48,49)”
- Page 16, line 461: Replace “abortus” with “aborted” or “abortion”
- Page 17, line 476: Insert space between “birth” and “42”
- Page 17, line 502: Insert “,” after “ and therefore”

The manuscript is good for publication after the minor corrections are conducted.
