# Supplementary material for: Seroprevalence and related risk factors of Brucella spp. in livestock and humans in Garbatula subcounty, Isiolo county, Kenya
Source: PLoS Negl Trop Dis. 2023 Oct 16;17(10):e0011682. doi: 10.1371/journal.pntd.0011682 (PMC10602376; doi:10.1371/journal.pntd.0011682)
Supplement: Attachment — Submitted filename: Bruc_Seroprev_August2023_Response to reviewer questions.docx [file pntd.0011682.s006.docx]

**Reviewer 1**

1. line 17- remove repeating ‘brucellosis is’

This has been addressed in line 18.

2. Line 37- Indicate the OR for each species

Thank you for pointing out this. The OR for cattle and camels have been indicated in lines 39.

3. Line 172- What if the herd had less than 5 animals per spp?

For herds with less than 5 animals, all available animals were selected for the study. The following sentence was “In herds with fewer than five animals per species, all animals were selected” to clarify this point. Line 185-186

4. Line 174-Why exclude very sick animals?

We excluded very sick animals to avoid the additional stress of sampling these animals and to prevent an overestimation of disease prevalence. We therefore targeted apparently healthy animals.

5. Does it mean you included animals from above one month old? This needs to be looked at to exclude maternal antibodies.

Thank you for emphasizing this critical point. In our study, we included animals that were over one month old, potentially possessing maternal antibodies detectable by serological tests for up to 12 weeks of age. Importantly, there had been no livestock vaccination in the subcounty within the 12 months prior to our research. This scenario suggests that only infected pregnant animals could have transmitted these antibodies to their offspring. We included only 72 calves representing 0.03% of the sampled animals. We are confident that the impact of maternal antibodies on the outcomes is minimal. However, this has been acknowledged as a limitation in lines 518-520.

6. Line 189-192- Where was the final destination for human samples? Where were they analysed?

Thank you for bringing this up. Animal and human samples were analyzed at the International Livestock Research Institute (ILRI). “Testing of human samples was conducted at ILRI” has been added in line 204-205 to ensure clarity.

7. Line 216 (Laboratory Testing)-- Why two different ELISA kits for animals and human samples? Any reasons for selection of the test KIT used in livestock?

Thank you for this feedback. The IBL kit has been reported to have high sensitivity (100.0% CI 94.6–100.0) and specificity (98.6% CI 95.1–99.8) in humans (Fadeel et al 2011) https://www.microbiologyresearch.org/content/journal/jmm/10.1099/jmm.0.033381-0#tab2.

Additionally, the IBL Brucella kits have also been used in several studies in Kenya to test human samples thus providing scientific back-up for using this kit in humans.

<https://pubmed.ncbi.nlm.nih.gov/36467641/>

<https://journals.plos.org/plosntds/article?id=10.1371/journal.pntd.0009049>

<https://www.ncbi.nlm.nih.gov/pmc/articles/PMC4530738/>

The IDvet brucella kit Multispecies kits was selected due to its ability to detect anti-brucella antibodies in Bovines, caprine, camelid and ovine samples which were sampled in our study. High sensitivity and specificity have been reported for this kit as well (<https://www.ncbi.nlm.nih.gov/pmc/articles/PMC10028928/>). Further, the selected IDvet kit is based on an indirect ELISA assay using purified brucella-LPS (lipopolysaccharide) antigen that has been recommended by the OIE (WOAH) to be highly sensitive for the detection of anti-Brucella antibodies. (Manual of Diagnostic Tests and Vaccines for Terrestrial Animals 2013, Chapter 2.4.3).

8. Line 218- cancel word against

The wors ‘against’ has been cancelled- on line 231

9. Line 266/267- How did you exclude maternal antibodies in these age groups?

Maternal antibodies in young animals were not excluded. This has been acknowledged in the as a limitation in line 518-520

10.- Line 523- (Limitations)- I would also include the limitation of the testing kits used as it aimed at detecting only IgG. It is probably that you missed a number of early infected individuals. I am not sure if vaccination has never been a practice in the study area, otherwise the prevalence could as well be an over estimation as iELISA can not distinguish vaccination from natural infection

Thank you for your insightful comments and suggestions. The limitations of the testing kits used are a valid concern. We employed kits to detect IgG and there is a possibility that early infected animals with predominantly IgM response may not have been captured. We acknowledge this as a limitation in our study and provide text in the limitation section line 520-526

Regarding vaccination practices in the study area, there was no livestock vaccination within the 12 months preceding our research, but we acknowledge that historical vaccination can have an impact on the prevalence estimates.

**Reviewer 2**

11. Can the authors give reasons why Rose Bengal test (RBT) was not used for screening?, or for adaptation of brucellosis testing in series which was not performed. How would the authors explain the elimination of false positives because of the widely used serologic ELISAs for the detection of brucellosis which is very high, to the extent that some laboratories apply not only the CFT, but parallel with it, the classic Rose Bengal (RBT) or/and serum agglutination (SAT) tests for confirmation. There is the possibility of cross-reaction of Gram-negative bacterium species or other unknown organisms or molecular antigens that are generally considered as contributing in false positives using ELISA, how would the authors clarify this in this study.

Serology alone can only be a presumptive test since other pathogens (for example, Y. 337 enterocolitica, O:9) can cross-react with the tests, thereby leading to false-positive results.

Thank you for this insightful question.

We opted for IgG serology due to its widespread use and established effectiveness in detecting Brucella-specific antibodies. The decision was guided by the availability of resources and the aim to maintain consistency with previous studies in the region. While we acknowledge the benefits of employing a testing series with RBT or SAT for confirmation, our study's objectives centred around estimating the seroprevalence of brucellosis. Thus, we focused on IgG serology for its practicality and comparability with existing data. Furthermore, several studies have demonstrated a high level of agreement in detecting *Brucella* antibodies when both RBT and ELISA are used.

<https://bmcvetres.biomedcentral.com/articles/10.1186/s12917-021-03036-z>

<https://www.ncbi.nlm.nih.gov/pmc/articles/PMC7943594/>

Nonetheless, we agree that the use of RBT would have greatly increased the confidence of our results, and this has been acknowledged in the limitation section on lines 520 to 526

12. Can the authors elaborate more on the vaccination status or strategies in the study areas, as seropositivity could have been due to reaction to vaccination.

Regarding vaccination practices in the study area, there was no livestock vaccination within the 12 months preceding our research, but we acknowledge that historical vaccination can have an impact on the prevalence estimates.

**13. Abstract**

- Page 2, line 31: Remove “high” and replace with “an”
- Page 2, line 36: insert “anti-brucella” before antibody detection
- Page 2, line 44: change “*spp*” in italics to “spp.”
- Page 2, line 45: put :”the” before “disease spread”
- Page 2, line 51: Replace “Brucella” with “*Brucella*” in italics
- Page 2, line 63: Put a space between “globally” and “(1,2).”
- Page 3, line 69: Put a space between “species” and (6)
- Page 3, line 69: Replace “consuming” with “consumption of”
- Page 3, line 87: Put space between “complications” and “(14)”
- Page 3, line 88: Remove “-“ between “water-contaminated”
- Page 3, line 89: Replace “like” with “such as”
- Page 3, line 94: Insert coma “,” after developed countries and insert “it” before “is”
- Page 3, line 95: Remove full stop “.” after “Middle East”
- Page 3, line 97: Put space between “numbers” and “(20)”
- Page 3, lines 102 – 103: the authors may wish to use “%” in place of “per cent”
- Page 3, line 103: Insert space between “livestock” and “(22 – 26)”
- Page 3, line 105: Replace “one-health” with “One-Health”
- Page 3, line 105: Replace “includingntegrated” with “including intergrated”
- Page 3, line 108: insert “.” After “spp”

All editorial suggestions in Abstract have been incorporated the revised manuscript.

**14. Materials and methods**

- Page 4, line 116: Remove “sought and”
- Page 4, line 129, 134 and 135: Insert space between “459m”, “kilometre(31) and “inhabitants(31)”
- Page 5, line 148: Insert space between “size(n)”
- Page 6, line 176: Replace “these” with “them”
- Page 6, line 181 – 182: Can the authors using a uniform sign for degrees (°) ?? See 4-8°C differs from -20 ^0^C
- Page 6, line 184: Insert degree “°” between “20” and “C”
- Page 7, line 211: Replace “Lastly” with “Finally”
- Page 7, line 218: Remove “against” and insert space between “120” and “%”
- Page 8: Would the authors give the reference sensitivity and specificity for the serological tests used?

Thank you for this feedback. References for sensitivity and specificity has been provided. Two references have been added.

Matsumoto et al 2023

<https://www.ncbi.nlm.nih.gov/pmc/articles/PMC10028928/>

Fadeel et al 2011

<https://www.microbiologyresearch.org/content/journal/jmm/10.1099/jmm.0.033381->	0#tab2

- Page 8, line 238: Insert “C” after “37°) and insert space between “(pNPP)” and “substrate”
- Page 8, line 240: 37°C for 30 ???? what?

For 30 minutes

- Page 8, line 241: Replace “done” with “conducted”
- Page 8,line 249: Remove “)” after “OD”
- Page 8,lines 267 and 268: reduce spacing and insert appropriate spacing between numbers in brackets
- Page 8, line 289: Replace “Lastly” with “Finally”
- Page 8, line 290: Insert “, this” before “was”

All editorial suggestions in Materials and Methods have been incorporated in the revised manuscript.

**15. Results**

- Page 9, line 299: Insert “anti-*Brucella”* before “antibodies”
- Page 10, line 312: Remove “,” after “spp.”
- Page 11, line 334: Insert “95% CI:” before “84.0-89.0”
- Page 11, line 340: insert “who” after “those”
- Page 11, line 344: Romove “.” After “(95% CI: 39.9-54.9)” and insert “.” After (Table).
- Page 13, line 371: Replace “unboiled” with “raw milk”
- Page 13, line 372: Replace “boiled” with “pasteurized”
- Page 14, line 403: Replace “unboiled” with “raw milk”
- Page 14, line 404: Replace “Brucella” with “*Brucella*” in italics
- Page 14, line 406: Replace “with not” with “without”
- Page 14, line 407: Remove “<” before “0.01”
- Page 15, line 414: Replace “risk factors associated” with “associated risk factors”
- Page 15, lines 424 -426: Repetition “The high seroprevalence highlights the high endemicity of the disease in our study area due to the constant exposure of study participants to infected animals and animal products.”

This line was removed

- Page 16, line 434: Insert “,” after “Although”
- Page 16, line 458: Insert space between “infection” and “(48,49)”
- Page 16, line 461: Replace “abortus” with “aborted” or “abortion”
- Page 17, line 476: Insert space between “birth” and “42”
- Page 17, line 502: Insert “,” after “ and therefore”

All editorial suggestions in the Results section have been incorporated into the revised manuscript.

**Reviewer 3**

16. Title- Your title would be better if it is rearranged as Seroprevalence and related risk factors of Brucella spp. in livestock and humans in Garbatula subcounty, Isiolo county, Kenya.

Thank you for your valuable feedback. We appreciate your suggestion to rearrange the title as "Seroprevalence and related risk factors of Brucella spp. in livestock and humans in Garbatula subcounty, Isiolo county, Kenya." We agree that this revised title provides a clearer and more concise description of our research focus. Lines 1-2

17. Line 88 - Transmission routes in livestock is not discussed enough since there are several undiscussed methods of transmission.

Thank you for this feedback. I have indeed taken it into consideration and included additional information about various modes of transmission in the manuscript. Lines 97-100

18. Line 102- Please write as percent rather than per cent.

This has been changed from per cent to %

19. Line 103- Diagnostic options of the disease were not discussed. Since you have used one way of diagnostic approach which is sero-immunological study, this, and other diagnostic methods of are expected to be highlighted.

Thank you for this comment and guidance. We agree on the need to highlight the other diagnostic approaches in the manuscript. We have highlighted the diagnostic options in lines 85-90

20. Line 109/110- This objective was hidden or kept secret in the title of the manuscript. To me it is important to give credit in the research topic.

Thank you for raising this point. This concern has been addressed in the revised title.

21. Line 124- How much you are sure that children under 13 years can understand about your study procedures? What is the importance of informing this age group?

Thank you for this comment. Indeed, not all children under 13 have the capacity to fully understand the study procedures. Part of the study procedure was obtaining blood, which caused brief pain. The line about explaining study procedures to children under 13 has been removed, Lines 136-137

22. Line 126- Are you sure that Garbatula can be representative of the county?? Why are you preferred to use Isiolo County in the topic rather than Garbatula? Are you sure that they are similar in terms of agroecology or diseases distribution?

Thank you for this comment. We have addressed this comment by revising the title of the study to reflect the study site, Line 1-2

23. Line 137/138-‘good accessibility and water security’ There are no criteria for purposive sampling technique, rather they are preferably used for convenience sampling. Therefore, it requires rearrangement probably by removing the phrases.

Thank you for this guidance. The phrases on reasons for selection of Garbatula Subcounty has been removed. Line 149

24. Line 149- Sample size calculation- By what mechanisms you increased your sample to 2157?? What kind of formula or strategy you used to increase too that much? It has to be clarified.

Thank you for this feedback. The study targeted both humans and animals within households. Due to the lower number of humans present in households, we expanded our visits to more households to attain the desired sample size. Consequently, this led to an increased number of livestock samples. The inclusion of these additional 300 animal samples enhances the statistical power of our study.

25. Line 150/152- ‘no reliable references- This means that there are certain research findings but they are not reliable. Have you done reliability test?? How did you confirm that they are not reliable?

Thank you for pointing this out. I wrongly used the term ‘reliable reference’ in this context. I have therefore removed it and replaced it with ‘studies’. Line 162

26. Line 159-1844 animals-- How did you distribute this number to sampling unit of animal species involved in the study? Are they really proportionally distributed?

We arrived at 20 animals considering a maximum of 5 animals per species. This is explained in line 183-184

27. Line 174—'animals under one month’ - Have you checked for vaccination history of the animals? Most commonly animals less than six months of age are not selected for seroprevalence study, because false positive result might be detected.

Thank you for this feedback. There was no vaccination of animals 12 month prior to our study, but we acknowledge the implication of historical vaccination on the results of our study. This has now been addressed in the limitation section, Line 518-520

28. Line 191- ‘procedures for extracting serum’- Where did you demonstrated the human samples? Clarify it.

The procedures for extracting serum from human samples were identical to those used for animals. We included a statement in line 203-205 that states ‘The procedures for extracting serum, storing and transporting samples were identical to those used for animal samples’.

29. Line 208-210- ‘animal age groups’ - What were your criteria for these categories? How did you categorize in such way? was it arbitrarly taken? It must be clarified.

Thank you for pointing this out. The criteria and categories were based on previous studies in a similar pastoralist setup.

<https://journals.plos.org/plosntds/article?id=10.1371/journal.pntd.0010214>

<https://journals.plos.org/plosntds/article?id=10.1371/journal.pntd.0007506>

30. Line 218- ‘IgG’- The first word of any sentence cannot be abbreviated?

The short form of *Brucella* species was written as ssp.

32. Line 281- ‘p-value higher than 0.20 from the univariable analysis--- Is it higher than or lower than 0.20? If the p- value higher than 0.2 were selected for multivariable analysis?

Thank you for pointing this out. I acknowledge that this was a mistake and I have corrected to read as ‘lower than.’ Line 294

33. Line 313- ‘ Seroprevalence results’ -- The table is self-explanatory.\yo No need of writing everything about the table so that it may not be redundant. Where did you discuss the value of chi square?? I could not find it from the table. It is informal to discuss figures that is not explained in the table. You have to either add the value in to the table or remove from here.

Thank you for this guidance. We have now limited our results to what is presented in the table. Therefore, results from the Chi square tests have been removed. Additionally, the write-up has been summarized as advised. Line 328-344 for livestock data and 351 to 364 for human data.

34. Line 316/317- ‘cattle at 13.2 % (95 CI: 9.3-317 17.1), goats at 13.1% (95 CI: 11.1-15.3)’ - These values are interchanged and must be correctly written.

Thank you for pointing this out. The values had been interchanged but have now been correctly placed. Table 1

35. Line 329- Table 1- Table 1. Univariable mixed-effects logistic regression analysis of the factors associated with livestock brucellosis seropositivity. Sex- Why did you prefer higher prevalence as a reference category? Animal species -- Why did you prefer higher prevalence as a reference category?

Regarding the selection of reference categories, we opted for females as the reference for both human and animal samples. The decision to use goats as the reference category was driven by the higher number of samples (N) available for this group. For variables such as age group, educational level and herd size, we chose an ordinal scaling approach to determine our reference category. This choice was made to ensure ease of comprehending the data. In cases where variables were characterized by 'YES' and 'NO' responses, we designated the 'NO' category as the reference. This decision was grounded in the anticipation that potential risks would likely manifest in the 'YES' category, thus aiding in the interpretation of outcomes. In relation to location, we established Kinna as the reference category for both human and animal subjects.

36. Column ‘n’-- What does this n represent? Is it the number of sample or number of positive animals? Both should be presented separately to avoid ambiguity among your readers.

Thank you for this. We have clarified. ‘N’ is the total number of sampled animals and humans per category. We have now corrected this to the capital N and added a footnote to explain what it means in all tables.

37. Column ‘Seroprevalence % (95% CI)’- If this column is animal level seroprevalence, where are the values of the herd level seroprevalence results?? It must be kept in the table.

Thank you for pointing this out. The herd level seroprevalence results have now been added to table 1.

38. Line 333-344--- I could not find this result from the table. where do they come from? Too long paragraph. The table is independent enough to explain the result.

Thank you for this feedback. We have removed all results from the Chi square test and summarized our results further as guided.

39. Line 345-Table 2- Pls finish it on the single page only. Table should be completed on one page as much as possible. Column ‘n’-- What does this n represent? Is it the number of samples or number of positive animals? Both should be presented separately to avoid ambiguity among your readers. To be addressed.

Thank you, for this feedback. The guidance is well received. The tables will be placed in a new page. The column ‘n’ has been corrected to ‘N’ to means total number of sampled animals and humans per category.

40. Variable ‘Consumed milk (boiled or unboiled)- Where is the reference category? pls identify it.

The reference category has been identified as guided, in Table 2

41. ‘Handled animals’ and ‘handled manure’- Why you did not make uniform reference category? Somewhere you used higher prevalence but at majority of your reports you used the lower prevalence as a reference category. But, whatever it is it has to be uniform.

For variables with ‘YES’ and ‘NO’ such as ‘handled animals’ and ‘handled manure’ the NO category was the preferred reference category since the risk is expected to be in the YES category. Table 2

42. ‘Handled raw meat’ -- How these prevalence could be more than 100%? Try to check all?

The prevalence for each response to the variable is independent as a proportion of positive cases that handled raw meat or did not handle raw meat. This means that it could be more than 100%. We have made further clarity by changing the column ‘n’ in both tables to refer the total number of sampled animals and humans. Table 2

43. Line 348-results from univariable analysis- I am not clear with this paragraph. To say it is a result it was discussed in the above (why it is found important to repeat it?) Remove this paragraph from here and incorporate in to discussion part.

Thank you for pointing this out. This guidance has been considered and this section has been removed.

44. Line 350/351- From the prevalence of the from the goat is higher than that of cattle.

This section has been removed.

45. Line 363-365- Remove this paragraph from here and incorporate in to discussion part.

This guidance has been considered and therefore the whole section has been removed.

46. Line 366-374- Like the above paragraph, this paragraph is redundant. Remove this paragraph from here and incorporate in to discussion part.

The feedback has been considered and the paragraph was removed from these sections.

47. Line 377- multivariable analysis- Too long paragraph. Minimize here and discuss it in detail under discussion part.

Thank you for this feedback. We have retained the text in the multivariable analysis section but greatly reduced the univariable section.

48. Line 377 Why did you include age, sex and animal species and even herd size?? Ok, incase if you may wrongly selected these variables, Why you did not select history of abortion and sampling location to make your choice uniform? Hence, you have to rearrange the criteria of your selection for multivariable analysis. Criteria must P-value < 0.2 rather than >0.2. Based on the recommendation given by Trustfield (2018), P-value less than 0.2, 0.25 or 0.3 can be selected. But you have to know that you may probably miss significant factor when 0.2 is selected.

Thank you for this comment. We selected variable with P value below 0.2 (this was corrected in the text). We included sampling locations, herd size, animal age, animal species and sex. Both tables 3 and 4 only present the significant variables.

49. Line 379 – ‘animal age, sex, specie and herd size’- Why you did not select sampling location to make your choice uniform since they have P-value <0.2?

We included sampling location, but it was not significant at multivariable analysis.

50. Line 410-Table 4 (human multivariable analysis)- Why did you select only these variable while there are many other variables which fit the criteria of your selection? How did you exclude them?

We selected all variables that had a p value <0.02 but only presented variables that were significant in multivariable analysis. This was to reduce the size of the tables.

51. Line 417- ‘seroprevalence of 54% (95%CI: 50.2-58.0) and household seroprevalence of 418 86.4% (95% CI: 84.0 -89.0).’- This figure was not presented in the table.

We have included these figures in the tables 1 and 2

52. Line 427- By assuming that the presented result in Table is animals level seroprevalence, I did not see herd level seroprevalence. It has to be clearly presented.

Thank you for this feedback. This has been addressed and we have included the herd seroprevalence in Table 2

53. Line 429-‘higher than reported’ - It would be very good if you add the specific figure of the other related or unrelated reports.

Thank you for this feedback. We have included the seroprevalence data for the other comparative studies in lines 420-421

54. Line 431/432-‘ … followed closely by goats and cattle, while sheep had the lowest seroprevalence’-- This sentence requires rearrangement.

This sentence has been rearranged to read “The seroprevalence varied by species, with camels having the highest seroprevalence, followed closely by goats and cattle, while sheep exhibited the lowest seroprevalence.” Lines 421-423

55. Line 450—‘…found to be five time’ - From where did you get this value? The one given on the table is 7.6 not 5.

Thank you for this feedback. We have wrongly used the odds ratio from the Univariable analysis. The statement has been corrected to read “Adult animals were found to be more than seven times as likely as calves to be exposed to brucellosis” on line 421

56. Line 500- Borana- not italic

This has been corrected in the text Line 491
